# Atractylenolide III Ameliorates Bile Duct Ligation-Induced Liver Fibrosis by Inhibiting the PI3K/AKT Pathway and Regulating Glutamine Metabolism

**DOI:** 10.3390/molecules28145504

**Published:** 2023-07-19

**Authors:** Yan Wang, Kun Shi, Jiyuan Tu, Chang Ke, Niping Chen, Bo Wang, Yanju Liu, Zhongshi Zhou

**Affiliations:** 1 College of Pharmacy, Hubei University of Chinese Medicine, Wuhan 430065, China; wyy1975921@163.com (Y.W.); s1062033640@163.com (K.S.); 3070@hbtcm.edu.cn (J.T.); kczzz@foxmail.com (C.K.); cnp15207123792@163.com (N.C.); 2 Center for Hubei TCM Processing Technology Engineering, Wuhan 430065, China; 3 Hubei Institute for Drug Control, NMPA Key Laboratory of Quality Control of Chinese Medicine, Hubei Engineering Research Center for Drug Quality Control, Wuhan 430075, China; wang_bo1986@hotmail.com

**Keywords:** Atractylenolide III, bile duct ligation, liver fibrosis, PI3K/AKT, glutaminase

## Abstract

Liver fibrosis is one of the leading causes of hepatic sclerosis and hepatocellular carcinoma worldwide. However, the complex pathophysiological mechanisms of liver fibrosis are unknown, and no specific drugs are available to treat liver fibrosis. Atractylenolide III (ATL III) is a natural compound isolated from the plant *Atractylodes lancea* (Thunb.) DC. that possesses antioxidant properties and the ability to inhibit inflammatory responses. In this study, cholestatic hepatic fibrosis was induced in mice using a bile duct ligation (BDL) model and treated with 10 mg/kg and 50 mg/kg of ATL III via gavage for 14 days. ATL III significantly reduced the liver index, lowered serum ALT and AST levels, and reduced liver injury in bile-duct-ligated mice. In addition, ATL III significantly attenuated histopathological changes and reduced collagen deposition. ATL III reduced the expression of fibrosis-related genes α-smooth muscle actin (α-SMA), Collagen I (col1a1), Collagen IV (col4a2), and fibrosis-related proteins α-SMA and col1a1 in liver tissue. Using RNA sequencing (RNA-seq) to screen molecular targets and pathways, ATL III was found to affect the PI3K/AKT singling pathway by inhibiting the phosphorylation of PI3K and AKT, thereby ameliorating BDL-induced liver fibrosis. Gas chromatography–mass spectrometry (GC-MS) was used to evaluate the effect of ATL III on liver metabolites in BDL mice. ATL III further affected glutamine metabolism by down-regulating the activity of glutamine (GLS1) and glutamine metabolism. ATL III further affected glutamine metabolism by down-regulating the activity of glutaminase (GLS1), as well as glutamine metabolism. Therefore, we conclude that ATL III attenuates liver fibrosis by inhibiting the PI3K/AKT pathway and glutamine metabolism, suggesting that ATL III is a potential drug candidate for treating liver fibrosis.

## 1. Introduction

Liver fibrosis is a lesion caused by chronic liver injury and accumulation of the extracellular matrix (ECM) due to various factors, including chronic hepatitis C virus, hepatitis B virus infection, alcoholic fatty liver, and non-alcoholic steatohepatitis [1,2]. The imbalance of collagen as well as ECM synthesis and degradation after liver injury leads to excessive production and deposition of fibrous tissue in the hepatic lobules and confluent areas [3]. Liver fibrosis further progresses to cirrhosis and hepatocellular carcinoma, which is a serious health hazard; thus, it is of great clinical importance to study drugs able to improve liver fibrosis.

Transcriptomics refers to the quantitative assessment of all coding and non-coding RNA transcripts and reflects the transcriptional activity of a cell [4]. Transcriptomics has been widely used and has contributed significantly to our understanding of the pathophysiology of liver fibrosis. With rapid improvements in available technologies and data analysis, transcriptomics is now routinely used to identify potential mechanistic pathways to determine the impact of drug candidates for the treatment of liver fibrosis [5]. Metabolomics refers to the study of small molecules and metabolites such as amino acids, fatty acids, and carbohydrates. During the development of liver fibrosis, intrinsic metabolic reprogramming of hepatic stellate cells and hepatocytes has emerged as a key determinant of the cellular phenotypic transition that occurs in conjunction with chronic liver injury and is now recognized as a potential therapeutic target for liver fibrosis [6]. Understanding how hepatic metabolites affect liver fibrosis is critical for predicting the anti-fibrotic effects of targeting candidate pathways.

Liver fibrosis is characterized by metabolic abnormalities. Its development is accompanied by a series of disordered energy metabolism processes such as glucose metabolism, lipid metabolism, and glutamine metabolism [7], and its progression is due to enhanced glucose metabolism and enhanced fatty acid oxidation [8,9]. Glutamine exerts an important metabolic function in the synthesis of non-essential amino acids, maintenance of important mitochondrial metabolites, and production of antioxidants that counteract ROS. Glutamine catabolism increases in response to liver damage due to the increased expression of the genes regulating it, resulting in increased glutamine consumption. ATL III can significantly enhance the glucose uptake capacity of skeletal muscle cells C2C12 and increase mitochondrial mass and total ATP content in myotubes to promote energy metabolism [10]. Therefore, the investigation of the potential involvement of ATL III in the regulation of energy metabolism during liver fibrosis may result in a potentially effective therapy.

ATL III is the main active component of the plant *Atractylodes lancea* (Thunb.) DC. (https://mpns.science.kew.org/ (accessed on 7 June 2022)) [11]. This compound possesses several beneficial effects such as anti-inflammatory, antioxidant, and anti-viral properties, as well as gastrointestinal regulation and neuroprotection [12,13]. ATL III regulates multiple signaling pathways including those involved in reducing lipid deposition in HepG2 cells by the activation of the AMPK/SIRT1 signaling pathway downstream of adiponectin receptor 1 to treat non-alcoholic fatty liver disease [14]; exerts antidepressant and anxiolytic-like effects by inhibiting hippocampal neuronal inflammation [15]; attenuates lung fibrosis by alleviating oxidative stress through the Nrf2/NQO1/HO-1 pathway [16]; and finally, it attenuates heterogeneous lung fibrosis by regulating the PI3K/AKT/mTOR signaling pathway attenuated by isoflurane-induced neuronal damage in the rat hippocampus [17]. In addition, the great potential of ATL III in the treatment of hepatic fibrosis was recently confirmed using the cellular heat transfer technique and microcalorimetry [18]. Therefore, the in-depth investigation of the effect of ATL III in the treatment of fibrosis and its molecular mechanism is of utmost importance to provide an experimental basis for the development of a new ATL III drug.

The aim of this study was to investigate the potential role of ATL III in ameliorating liver fibrosis, as well as in the regulation of liver metabolism and, if so, its mechanisms of action. A bile duct ligation model was performed to obtain liver fibrosis in mice. Their serum biochemical parameters and pathological examination were performed to assess the effect of ATL III on liver injury. Transcriptome and metabolome analyses were also performed to evaluate the potential targets. Our study demonstrated that ATL III alleviated liver fibrosis by inhibiting the PI3K/AKT signaling pathway and decreasing glutamine metabolism in the liver. Thus, ATL III might represent a potential candidate in the treatment of liver fibrosis.

## 2. Results

### 2.1. ATL III Inhibits BDL-Induced Liver Injury

**Figure 1:**
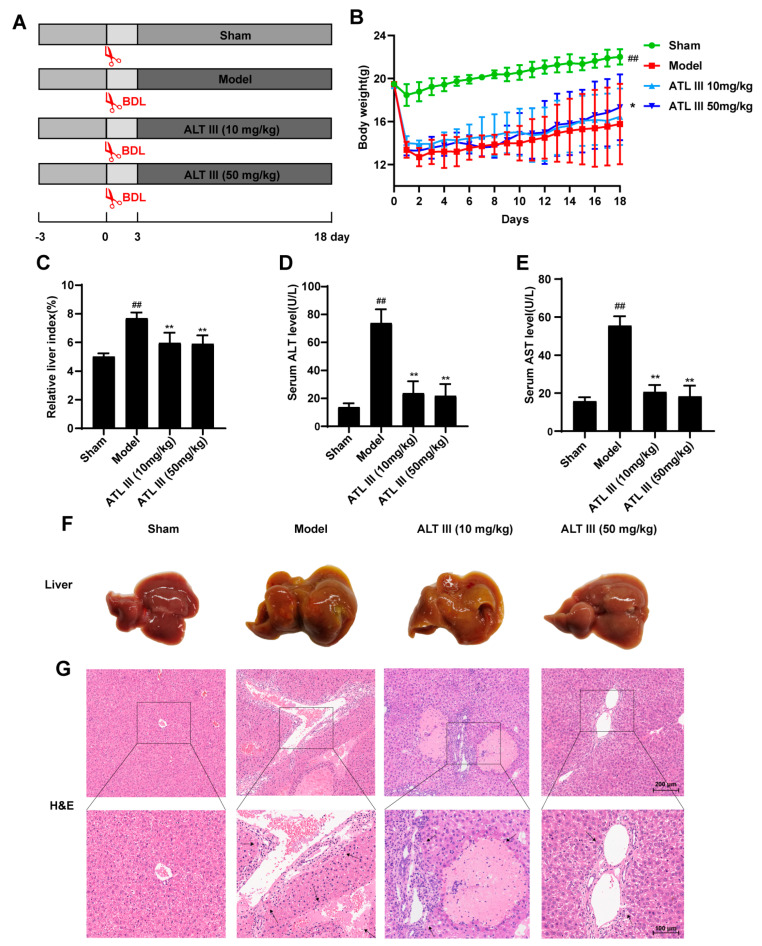
ATL III inhibited BDL-induced liver injury. (**A**) Schematic diagram of the animal experimental design. (**B**) Body weight. (**C**) Liver index. Levels of (**D**) ALT and (**E**) AST. (**F**) Image of liver tissue at low magnification. (**G**) Representative H&E staining images of liver tissue sections. Results are expressed as mean ± SD, *n* = 3. ^##^ *p* < 0.01 compared with the sham group. * *p* < 0.05, ** *p* < 0.01 compared with the model group.

ATL III at both 10 mg/kg and 50 mg/kg significantly reduced the extent of liver injury. The effect of ATL III on BDL-induced liver fibrosis was assessed by the comparison of the ATL III treatment group with the sham and model groups. We performed BDL surgery on mice and the body weight of BDL mice decreased substantially compared with the Sham group and recovered after the administration of the ATL III treatment, which was significantly different compared with the model group (Figure 1A,B). The results showed that the liver index was significantly higher in the model group and significantly lower in the ATL III groups at both doses compared with the sham group (Figure 1C). A significant decrease in serum ALT and AST levels was observed after ATL III treatment (Figure 1D,E). The liver morphology showed a rougher surface in the liver of the model group with yellow spots compared with that in the sham group (Figure 1F). The livers of mice in both ATL III treatment groups had a smoother surface and fewer yellow spots compared with the model group. Extensive necrosis of the liver parenchyma, severe damage in the structure of the liver, and destruction of the liver lobular structure were observed in the model group, as well as hepatic ballooning, collagen deposition, and bile duct hyperplasia. In contrast, these lesions were significantly reduced in both ATL III treatment groups (Figure 1G). These results suggest that ATL III reduced liver injury caused by BDL potentially exerting a therapeutic role in liver fibrosis.

### 2.2. ATL III Ameliorates BDL-Induced Liver Fibrosis in Mice

**Figure 2:**
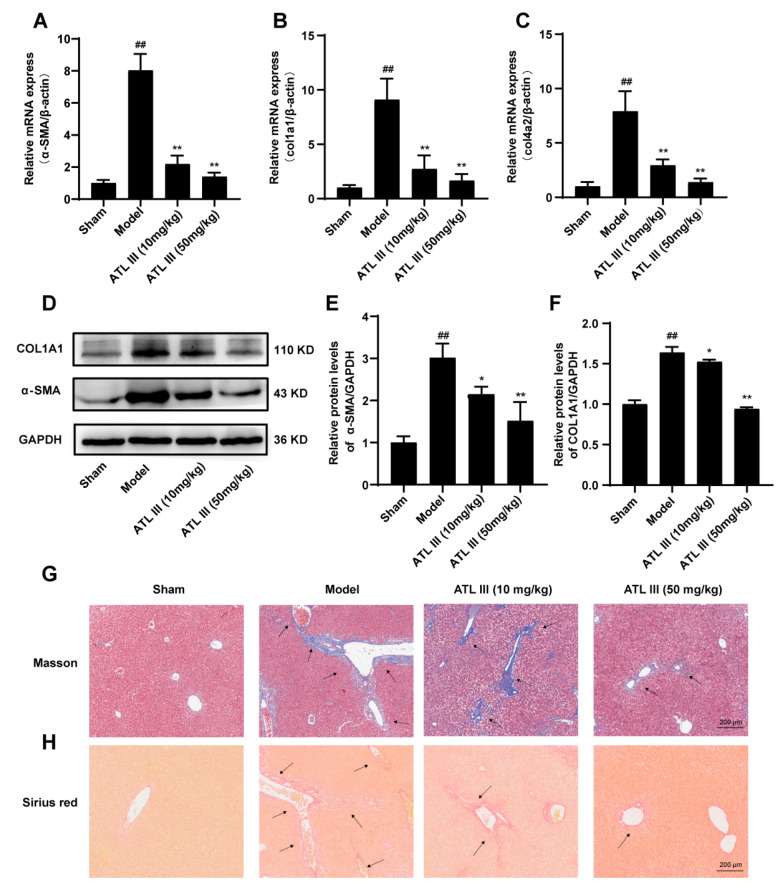
ATL III ameliorated BDL-induced liver fibrosis in mice. The mRNA expression of (**A**) α-SMA, (**B**) col1a1, and (**C**) col4a2 in the liver. Protein expression of (**D**) α-SMA and COL1A1 in the liver. (**F**) Protein expression statistics of (**E**) α-SMA and (**F**) COL1A1. (**G**) Representative MASSON-stained images of liver tissue sections. (**H**) Representative Sirius red-stained images of liver tissue sections. Results are expressed as mean ± SD, *n* = 3. ^##^ *p* < 0.01 compared with the Sham group. * *p* < 0.05, ** *p* < 0.01 compared with the model group.

**Figure 3:**
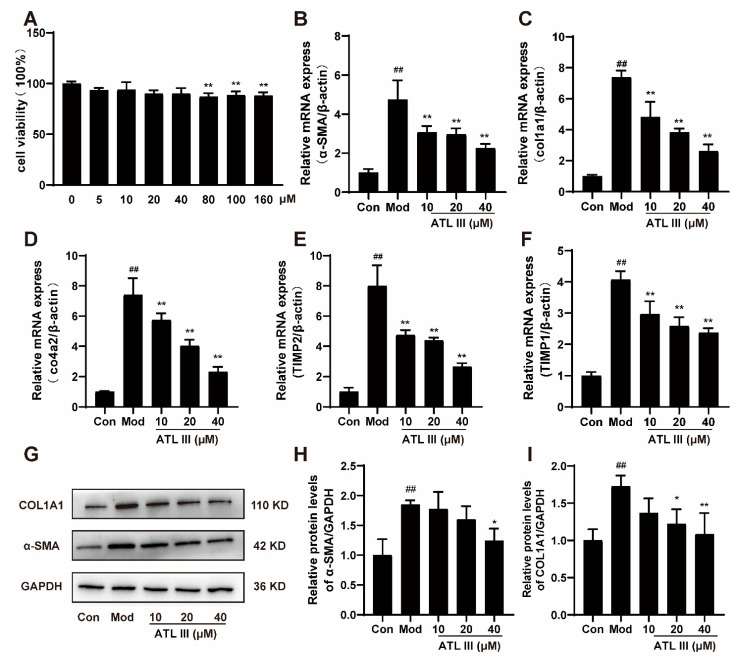
ATL III inhibits TGF-β1-stimulated LX-2 activation. (**A**) Effects of different concentrations of ATL III on the viability of LX-2. The mRNA expression of (**B**) α-SMA, (**C**) col1a1, (**D**) col4a2, (**E**) TIMP2, and (**F**) TIMP1 in LX-2. (**G**) Protein expression of α-SMA and COL1A1 in LX-2. Protein expression statistics of (**H**) α-SMA and (**I**) COL1A1. Data are expressed as the mean ± SD, *n* = 3. Data were analyzed using one-way ANOVA. ^##^ *p* < 0.01 compared with the Con group. * *p* < 0.05, ** *p* < 0.01 compared with the Mod group.

Next, the therapeutic potential of ATL III on liver fibrosis in BDL mice was evaluated. The results showed that ATL III inhibited the expression or the release of fibrosis-associated genes, such as the ECM proliferation-associated gene α-SMA, col1a1, and col4a2 (Figure 2A–C). Similarly, ATL III inhibited the protein expression of COL1A1 and α-SMA (Figure 2D–F). In addition, the collagen deposition in the liver to assess the extent of fibrosis revealed that the mice in the model group had a substantial amount of collagen deposition in the portal region of the liver, which even spread to the liver parenchyma. However, both the ATL III treatment groups showed only a small amount of collagen deposition near the liver portal vein, while no evident collagen deposition was observed in the liver parenchyma (Figure 2G,H). These results suggested that ATL III was effective in treating BDL-induced liver fibrosis. To further investigate the effect of ATL III in the treatment of liver fibrosis, we investigated the effect of ATL III on hematopoietic stem cells (LX-2) in vitro. The inhibitory effects of ATL III on LX-2 were studied at concentrations of 10, 20 and 40 μM selected according to the results of CCK8 experiments (Figure 3A). Compared with the model group, ATL III significantly decreased the expression of fibrosis-related genes α-SMA, cola1, col4a2, tissue inhibitor of metalloproteinase 1 (TIMP1), and tissue inhibitor of metalloproteinases 2 (TIMP2) (Figure 3B–F). And ATL III 40 μM significantly reduced the expression of α-SMA and col1a1 and inhibited the activation of LX-2 stimulated by TGF-β1 (Figure 3G–I).

### 2.3. ATL III Regulates the Differentially Expressed Genes and Pathway Enrichment Analysis in the Liver of BDL Mice

**Figure 4:**
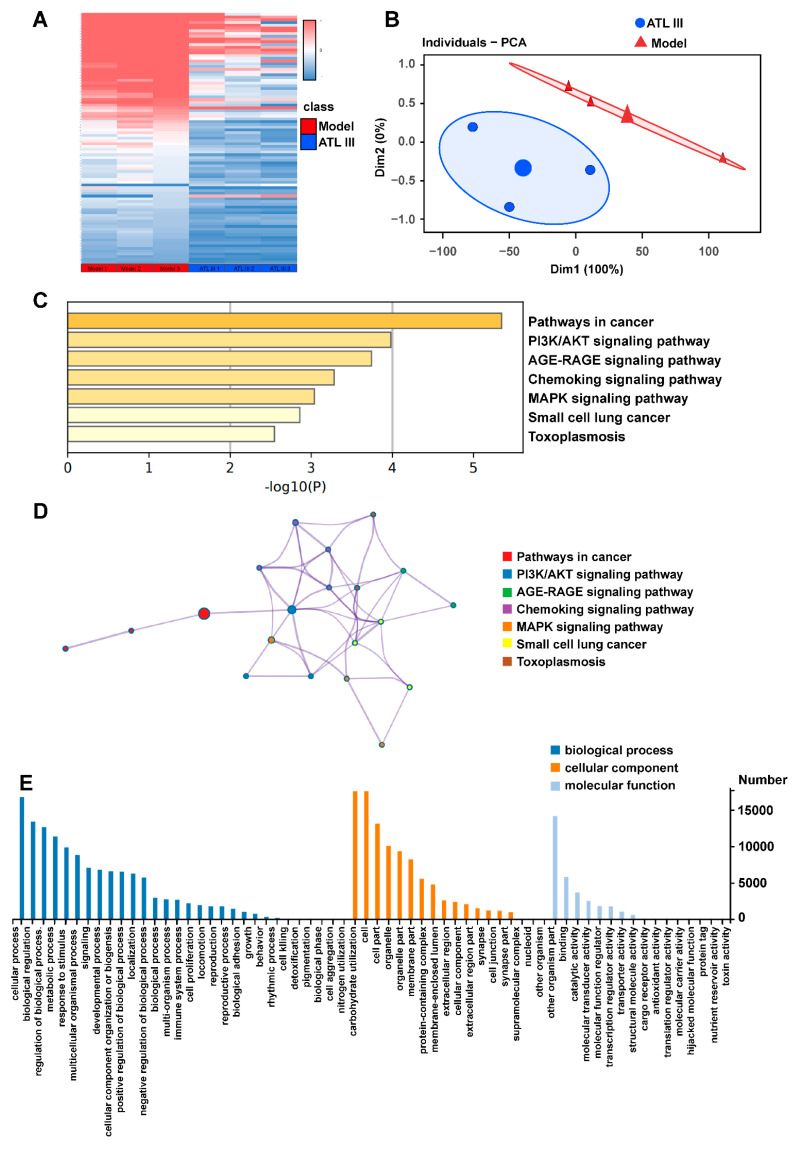
ATL III regulated the differentially expressed genes and pathway enrichment analysis in the liver of BDL mice. (**A**) Heat map of the mRNA associated with liver fibrosis in various groups of mice. (**B**) Principal component analysis (PCA) of RNA-seq data from the model and ATL III groups. (**C**) KEGG pathway enrichment analysis. (**D**) Enrichment GO. (**E**) GO annotation analysis showing the significant biological processes involved in the metabolic process of BDL mice treated with ATL III 50 mg/kg. *n* = 3 per group.

The dose of ATL III 50 mg/kg was chosen to study the mechanism of action of ATL III after it was clear that it had a pharmacological effect in alleviating liver fibrosis. Transcriptome analysis on the ATL III 50 mg/kg group and the model group was performed to understand the mechanism of action of ATL III and which genes were changed in vivo after ATL III treatment. The ATL III 50 mg/kg group was superior to the ATL III 10 mg/kg group in reducing intrahepatic fibroplasia-related protein expression. Therefore, the ATL III 50 mg/kg group was used to understand the mechanism of action. A significant separation between ATL III and the model group was found (Figure 4A), and the heat map showed that the two groups were well distinguished (Figure 4B). The KEGG pathway annotation and enrichment analysis showed that DEGs were mainly enriched in cancer, PI3K-AKT signaling pathway, AGE-RAGE signaling pathway, Chemokine signaling pathway, and MAPK signaling pathway (Figure 4C,D). Following that, KEGG signal pathway enrichment was performed and results showed that the DEGs highly enriched the biological process (Figure 4E).

### 2.4. ATL III Alleviates Liver Fibrosis by Inhibiting the PI3K/AKT Pathway

The progression of liver fibrosis is closely related to the internal cascade pathway phosphatidylinositol 3 kinase/protein kinase B (PI3K/AKT) signaling pathway [19]. The activation of the PI3K/AKT signaling pathway is involved in the formation of liver fibrosis and can be used as a target for various anti-fibrotic therapies [20,21]. In addition, the inhibition of the PI3K/AKT pathway accelerates the synthesis and degradation of ECM in the liver [22], significantly reduces carbon tetrachloride (CCl_4_)-induced collagen secretion, and improves liver fibrosis in mice [23,24].

**Figure 5:**
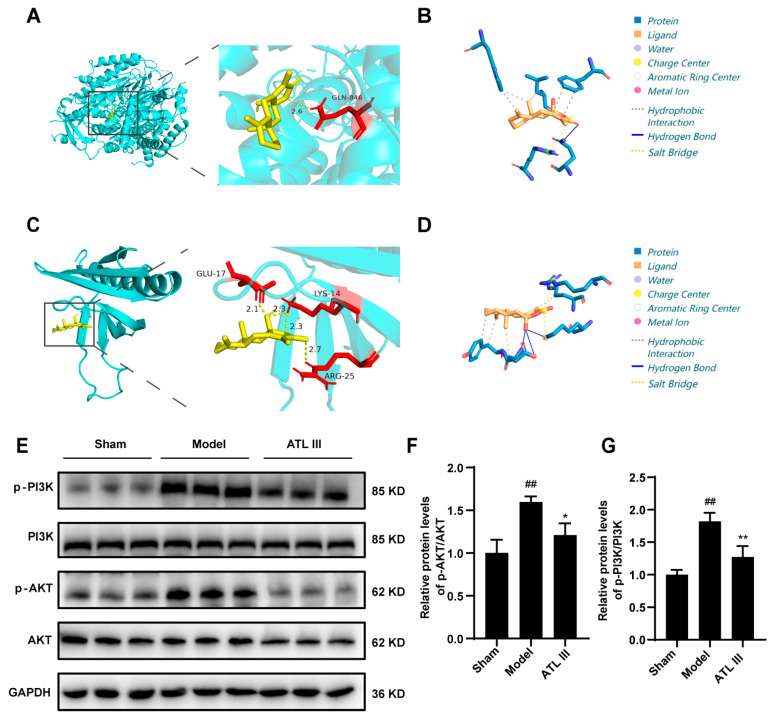
ATL III alleviates liver fibrosis by inhibiting the PI3K/AKT pathway. (**A**) Molecular docking of ATL III with the protein PI3K. (**B**) ATL III binding to the PI3K. (**C**) Molecular docking of ATL III with the protein AKT. (**D**) ATL III binding to the AKT. (**E**) Protein expression of p-PI3K, PI3K, p-AKT, and AKT in the liver. Protein expression statistics of (**F**) p-AKT/AKT and (**G**) p-PI3K/PI3K. Results are expressed as mean ± SD, *n* = 3. ^##^ *p* < 0.01 compared with the sham group. * *p* < 0.05, ** *p* < 0.01 compared with the model group.

Autoduck and PLIP were used to simulate the molecular docking of ATLIII with PI3K and AKT to evaluate the potential involvement of PI3K/AKT in the mechanism of action of ATL III in the reduction in liver fibrosis. ATL III formed a 2.6 Å hydrogen bond with the amino acid residue GLN-864 of PI3K with a minimum binding energy of −7.5 Kcal/mol (Figure 5A,B) (Table S2). ATL III formed a 2.1 Å hydrogen bond with the amino acid residue GLU-17, a 2.3 Å hydrogen bond with the amino acid residue LYS-14, and a 2.7 Å hydrogen bond with the amino acid residue ARG-25 of AKT with a minimum binding energy of −7.0 Kcal/mol (Figure 5C,D) (Table S3). The expression of p-PI3K, PI3K, p-AKT, and AKT proteins revealed that ATL III decreased the phosphorylation of PI3K and AKT compared with that in the model group (Figure 5E,F). The above results suggest that ATL III inhibited the development of liver fibrosis by suppressing the activation of the PI3K/AKT pathway.

### 2.5. ATL III Attenuates BDL-Induced Metabolic Disorders and Regulates Glutamine Metabolism in Mice

#### 2.5.1. Multivariate Statistical Analysis

**Figure 6:**
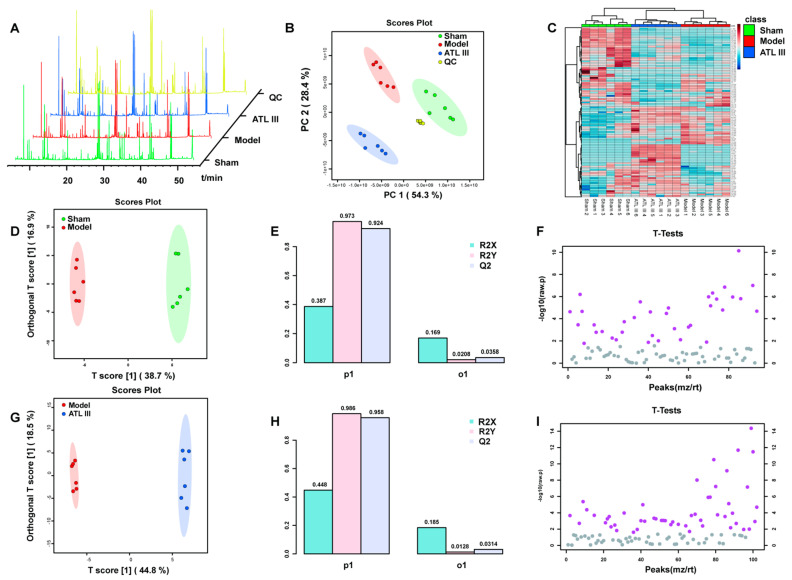
Multidimensional analysis of metabolite data. (**A**) TIC map of total ion currents of GC-MS metabolites in the liver of mice in each group. (**B**) PCA analysis of the liver GC-MS metabolites in mice in each group. (**C**) Heat map of metabolites showing the differences in the liver of mice in each group. (**D**) OPLS-DA analysis of metabolites in the sham and model groups. (**E**) OPLS-DA model parameters for the sham and model groups. (**F**) Student’s t-test analysis of metabolites in the sham and model groups. (**G**) OPLS-DA analysis of metabolites in the ATL III and model groups. (**H**) OPLS-DA model parameters for the ATL III and model groups. (**I**) Student’s t-test analysis of metabolites in the ATL III and model groups (*n* = 6).

The metabolism of the liver undergoes a series of disturbances during liver fibrosis. The results of GO annotation analysis also suggest that the biological processes included metabolic processes. The 50 mg/kg ATL III appeared to have the greatest effect on liver fibrosis; therefore, this dose was selected for use in our subsequent research. We used metabonomics to further verify the liver metabolite analysis of the sham group, the BDL group, and the ATLIII group. GC-MS was used to assess the effect of ATL III on primary metabolites in mouse liver, and the results are shown in Figure 6A. PCA performed on all samples showed a clear separation between the sham group, the model group, and the ATL III group, with a good aggregation of the principal components of the quality control samples, indicating the reliability of the experimental methods and data processing methods (Figure 6B). The heat map likewise showed that ATL III affected the production of many primary metabolites in the liver (Figure 6C). To further elucidate the ability of ATLIII to regulate metabolites, the differential metabolites and metabolic pathways associated with liver fibrosis were identified based on VIP > 1 in the orthogonal projection to latent structures square discriminate analysis (OPLS-DA) model combined with *p* < 0.05 in the Student’s t-test (Figure 6F,I). The established OPLS-DA model showed good discrimination between the sham and model groups. Similarly, the OPLS-DA model showed good discrimination between the ATL III and model groups (Figure 6D,G). In addition, the good predictive performance of the OPLS-DA model developed in this study was confirmed in 100 external experiments (Figure 6E,H). 

#### 2.5.2. Multivariate Statistical Analysis

**Figure 7:**
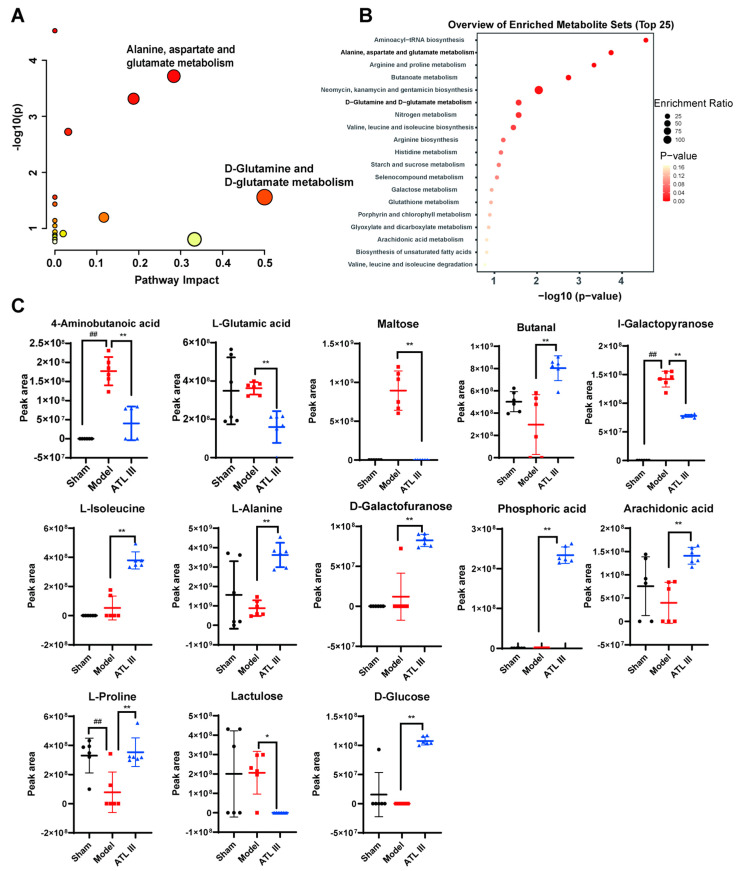
ATL III attenuated BDL-induced metabolic disorders and regulated glutamine metabolism in mice. (**A**) Pathway analysis of differential metabolites. (**B**) Enrichment analysis of differential metabolites. (**C**) Peak area comparison diagram of different metabolites. Results are expressed as mean ± SD, *n* = 6. ^##^ *p* < 0.01 compared with the sham group. * *p* < 0.05, ** *p* < 0.01 compared with the model group.

Compounds with VIP values > 1 and significant differences between groups (*p* < 0.01) in the OPLS-DA model were selected, and those meeting both conditions were considered as differential metabolites. ATL III changed the following primary metabolites compared with the model group: I-galactopyranose, L-alanine, L-isoleucine, phosphoric acid, L-proline, 4-aminobutanoic acid, butanal, D-galactofuranose, arachidonic acid, D-glucose, maltose, lactulose, and L-glutamic acid. The GC-MS peak areas of these compounds are shown in Figure 7C. KEGG signal pathway results showed that the top-ranked pathways were aminoacyl-tRNA biosynthesis; D-glutamine and D-glutamate metabolism; alanine, aspartate, and glutamate metabolism; and arginine biosynthesis (Figure 7A,B). The enrichment analysis showed that ATL III alleviated metabolic disorders and treated liver fibrosis by affecting multiple metabolic pathways. In contrast, D-glutamine and D-glutamate metabolism and alanine, aspartate, and glutamate metabolism were ranked in front of the bubble diagram, indicating that these two are very important. The above results suggested that the alleviating effect of ATL III on liver fibrosis might be related to glutamine metabolism.

### 2.6. Inhibition of BDL-Induced Liver Fibrosis by ATL III through the Inhibition of Glutaminase

**Figure 8:**
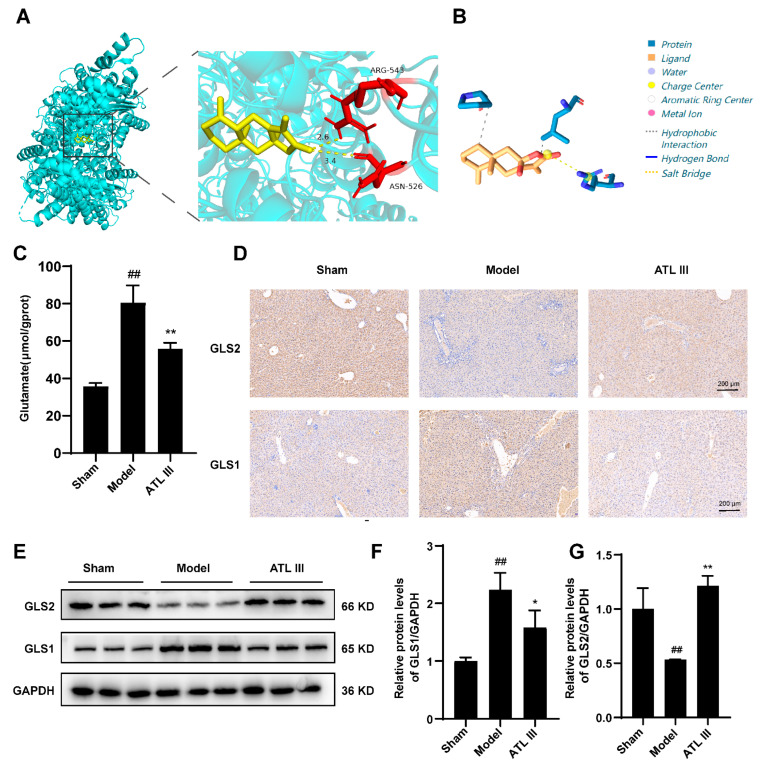
Inhibition of glutaminase by ATL III in BDL-induced liver fibrosis. (**A**) Molecular docking of ATL III with the protein GLS1. (**B**) ATL III binding to the GLS1. (**C**) Glutamate content in the liver. (**D**) Representative GLS1 and GLS2 immunohistochemical images of liver tissue sections. (**E**) Protein expression of GLS1 and GLS2 in the liver. Protein expression statistics of (**F**) GLS1 and (**G**) GLS2. Results are expressed as mean ± SD, *n* = 3. ^##^ *p* < 0.01 compared with the sham group. * *p* < 0.05, ** *p* < 0.01 compared with the model group.

Glutamine is catabolized in cells to glutamate by glutaminase (GLS), which has two main isoforms: one present in the kidney and many other tissues (called renal glutaminase or GLS1) and the other expressed in the liver (called hepatic glutaminase or GLS2). Autoduck and PLIP were used to simulate the molecular docking of ATL III with GLS1 to evaluate the potential involvement of GLS1 in the mechanism of action of ATL III in the reduction in liver fibrosis. ATL III formed a 2.6 Å hydrogen bond with the amino acid residue ARG-543 and a 3.4 Å hydrogen bond with the amino acid residue ASN-526 of GLS1 with a minimum binding energy of −7.8 Kcal/mol (Figure 8A,B) (Table S4). Disturbance in glutamine metabolism occurs during liver fibrosis, resulting in increased glutamate levels in the liver. The glutamate content in the liver was measured to evaluate the extent of glutamine metabolism disorder. The results showed that the glutamate content in the liver of the model group was significantly higher than that of the sham group and tended to decrease after the treatment with ATL III (Figure 8C). In addition, the expression of the glutamine metabolizing enzyme GLS in the liver was also examined, and GLS1 protein expression was significantly up-regulated in the model group compared with that in the sham group, and this trend was reversed by ATL III. On the contrary, the expression of GLS2 in the BDL mice showed a decreasing trend, and the drug intervention up-regulated the expression of GLS2 (Figure 8D–G). These results demonstrate that ATL III exerted a regulatory effect on metabolic abnormalities, especially glutamate metabolism in the model group.

## 3. Discussion

Liver fibrosis is a common pathological process in chronic liver injury such as liver cancer, liver failure, and cirrhosis, and this process is reversible; it has been considered an important aspect in the treatment of liver diseases [25]. Bile duct ligation is the most classic model of liver fibrosis; it consists of extrahepatic biliary obstruction, impaired bile secretion and excretion, excessive accumulation of bile in the liver, and intrahepatic vessels under the dual pressure of bile duct dilation and bile extravasation. Thus, it causes damage to hepatocytes and the organism [26]. Serum levels of ALT and AST are important and sensitive biochemical indicators for assessing liver function. The transaminases from the hepatocytes enter the bloodstream when the liver is damaged and the blood levels of ALT and AST increase. This study revealed that ATL III reduced the serum levels of ALT and AST in the model group and restored the damaged liver function. In addition, BDL caused a series of damages such as rough liver surface, dilated and ruptured intrahepatic bile ducts, extravasated bile, and broken ring of portal vein structure with significant collagen deposition, which were consistent with a previous report [27]. The abnormal synthesis of α-SMA and COL1A1 was found in the model group after liver damage compared with the sham group, and both are considered important molecular markers of liver fibrosis [28]. This trend was reversed by the ATL III treatment. Masson and Sirius red staining are considered as the gold standards for assessing collagen deposition, and both showed a decrease in collagen deposition around the portal vein after ATL III treatment. All these results support the ability of ATL III to attenuate BDL-induced liver fibrosis.

The regulatory pathways used by ATL III to exert its effects were assessed to further investigate the mechanism of action of ATL III in the treatment of liver injury. Our results show the presence of differentially expressed genes between the model group and the ATL III 50 mg/kg group. The differentially expressed genes were enriched in pathways of cancer, the PI3K-AKT signaling pathway, the AGE-RAGE signaling pathway, the chemokine signaling pathway, the MAPK signaling pathway, small cell lung cancer, and toxoplasmosis. Previous studies found that the accumulation of advanced glycosylation end products (AGEs) and activation of AGE receptors (RAGE) induce sustained oxidative stress in vascular tissues, and the inhibition of AGE/RAGE attenuates the progression of diabetic nephropathy [29,30]. MAPK signaling pathway is a major signaling pathway involved in cell surface to nucleus proliferation, and its inhibition induces antioxidant and anti-inflammatory effects, as well as attenuates carbon tetrachloride-induced liver fibrosis [31,32]. The PI3K/AKT signaling pathway is widely involved in the basic biological functions of cells, including cell proliferation, differentiation, apoptosis, and maintenance of cell physiology [33]; it is also involved in the formation of liver fibrosis by accelerating the progression of liver fibrosis through the promotion of cell proliferation and collagen synthesis [34]. PI3K/AKT inhibition effectively attenuates MMP-9 expression induced by tumor necrosis factor-like weak inducer of apoptosis (TWEAK) and reduces ECM deposition, thereby delaying the development of liver fibrosis [35]. Our results showed that the lowest binding energy of ATL III with PI3K was −7.5 Kcal/mol, respectively, revealing that it might be used as a potential target for PI3K and AKT. In addition, ATL III inhibited PI3K/AKT signaling pathway by inhibiting the phosphorylation of PI3K and AKT proteins, consequently treating liver fibrosis.

The demand for energy increases during the course of liver fibrosis. Thus, the liver’s metabolism is disturbed to meet the increased energy request. Our results showed metabolic differences between the model group and the ATL III 50 mg/kg group focused on several metabolisms including glucose metabolism, lipid metabolism, and glutamine metabolism. Although lipid metabolism and bile acid metabolism play important roles in the disease process of hepatic fibrosis, in this paper, non-targeted metabolomic analyses of mouse liver tissues showed that ATL III affects unsaturated fatty acid biosynthesis as well as the metabolism of glyoxylate and dicarboxylic acid in mouse liver; these two metabolic pathways associated with lipid metabolism in the liver were ranked relatively low in the bubble plot (−log10(p) < 1 with pathway effect < 0.1) and there were no significant differences, so we did not investigate lipid metabolism and bile acid metabolism profiles in depth. Furthermore, our compound pathway analysis emphasized D-glutamine and D-glutamate metabolism as the most important. Therefore, we conclude that ATL III may attenuate hepatic fibrosis primarily by affecting glutamine metabolism. Glutamine metabolism is involved in liver fibrosis, and it is divided into two steps. Glutamine is converted to glutamate by glutaminase (GLS), which is further metabolized to α-ketoglutarate, which participates in the TCA cycle and provides the ATP required for cellular metabolism. Glutamine content was revaluated in the liver of our mice, and the results showed that it was significantly higher in the model group compared with the sham group, decreasing after the ATL III treatment. This result demonstrated that ATL III affected glutamine metabolism in the liver of BDL mice. Glutamine metabolism produces glutamate, which is one of the sources of energy for the development of liver fibrosis, leading to its further deterioration [36]. Glutaminase expression in experimental models of chronic liver injury is increased in liver samples from both non-alcoholic steatohepatitis and advanced liver fibrosis patients [37,38]. Bile duct ligation causes obstruction of the extrahepatic bile ducts, which in turn causes bile duct dilatation and cholestasis. When the pressure in the bile ducts increases further, the intrahepatic bile ducts dilate and rupture, the intrahepatic blood vessels are compressed by both dilated bile ducts and bile extravasation, ischemia and necrosis occur in the hepatocytes, the hepatic stellate cells are activated and secrete a large amount of extracellular matrix, and a large amount of fibrous tissue proliferates, surrounding the hepatic lobules and scattering around the hepatocytes. Activation of hepatic stellate cells and the production and secretion of ECM require an adequate intracellular supply of ATP, which causes metabolic disorders in the liver, and glutamine metabolism from glutamine catabolism is the source of ATP for the differentiation of hepatic stellate cells into myofibroblasts. The inhibition of glutaminolysis with glutaminase inhibitors or glutamine deprivation reduces the worsening of liver fibrosis [39,40]. “Renal-type” glutaminase (GLS1) is a metabolic enzyme involved in glutamate catabolism [41]. GLS2 levels in the fibrotic liver are reduced and glutamine synthetase expression is lower, but GLS1 is up-regulated [42]. GLS1 is overexpressed in highly proliferating cancer cells to meet the demand for glutamine during the development of liver fibrosis. Our results further demonstrated that ATL III alleviated liver fibrosis by inhibiting GLS1-mediated glutamine catabolism and glutamine metabolism.

Alanine–serine–cysteine transporter type-2 (ASCT2) is a target of ATL III and it induces hematopoietic stem cell (HSC) senescence by reducing the metabolites of HSC, suggesting that ATL III may be involved in their regulation [18]. Our results suggest that ATL III inhibited the PI3K/AKT pathway and GLS1 in the glutamine metabolic pathway in the liver to alleviate liver fibrosis. The liver is the largest parenchymal organ of the body, consisting of hepatic parenchymal cells (hepatocytes) and non-parenchymal cells (including HSC, blood sinusoidal endothelial cells, and Kupffer cells). The biological function of the liver is due to the joint extensive interactions, precise regulation, and orderly division of the work of all these different cell types. Considering that HSCs represent only 1.5% of the total number of hepatic intrinsic cells in the whole liver [43], our result suggested that ATL III might affect not only HSCs but also other cells including hepatocytes. Hepatocytes are the cells most represented in the liver that originate from the primitive intestinal epithelium; they synthesize, degrade, transform, and store a variety of substances and secrete bile. Further analysis is required to establish which liver cells are affected by ATL III, and further exploration using magnetic beads to sort hepatocytes or the use of various pathways is needed to transform them into different subtypes by single-cell sequencing, to provide a basis for the development of new drugs to treat liver fibrosis.

## 4. Materials and Methods

### 4.1. Chemicals and Reagents

Atractylenolide III (purity > 98%, DSTDB001601) was purchased from Chengdu DeSiTe Biological Technology Co. Ltd. (Chengdu, China). Antibodies against GADPH (60004-1-Ig), α-SMA (14395-1-AP), COL1A1 (67288-1-Ig), AKT (10176-2-AP), p-AKT (66444-1-lg), and PI3K (#48184-1) were purchased from Proteintech (Wuhan, China). Antibodies against GLS1 (A3885) and GLS2 (A16029) were purchased from ABclonal Technology (Wuhan, China). Methanol (cat#67-56-1) was purchased from Merck (Darmstadt, Germany), acetonitrile (cat. #51101) was purchased from Thermo Fisher Scientific Inc. (Shanghai, China), and N-methyl-N-trimethylsilyl-trifluoro-acetamide (BSTFA, #FM05241802), methoxyamine hydrochloride (#BCBZ8981), and pyridine (#SHBK6453) were purchased from Sigma Aldrich, Co, St. (St. Louis, MO, USA).

ABScript III RT SuperMix was purchased from ABclonal Technology (RK20429, Wuhan, China) and Universal SYBR qPCR Master Mix from Vazyme Biotech Co., Ltd. (Q711-02, Nanjing, China). The Glutamic–pyruvic Transaminase (ATL) Assay Kit (cat. C009-2-1) and the Glutamic–oxalacetic Transaminase (AST) Assay Kit (cat.C010-2-1) were purchased from Nanjing Jiancheng Bioengineering Institute (China). A glutamate colorimetric test kit (E-BC-K118-M) was purchased from Elabscience Biotechnology Co., Ltd (Wuhan, China).

### 4.2. Animal Experiments

Forty SPF-grade C57BL/6 male mice, aged six weeks old and weighing approximately 18–22 g, were purchased from Liaoning Changsheng Biotechnology. All animals were housed at a temperature of 23 ± 1 °C, humidity of 65 ± 5%, subjected to a 12 h light/dark cycle, and fed adaptively for 3 days. All experiments were approved by the Animal Ethics Committee of the Hubei University of Traditional Chinese Medicine. The animal license is No. SYXK(E)2017-0067. The all-surgical procedure on the experimental animals met all ethical requirements and was approved by the Animal Ethics Committee of the Hubei University of Chinese Medicine (approval code: NO. 00273286, 10 December 2021).

Mice were anesthetized with an intraperitoneal injection of phenobarbital, cut along the midline of the abdomen, and the bile ducts were double ligated with non-absorbable surgical sutures [44]. All groups underwent bile duct ligation and the sham group underwent direct suturing without ligation. After 48 h of surgery, 40 male mice were randomly divided into 4 groups: (A) the sham group, (B) the model group, (C) the ATL III 10 mg/kg group, and (D) the ATL III 50 mg/kg group [45]. The ATL III was administered via oral gavage. ATL III was dissolved in 0.5% CMC-Na solution and gavaged once daily for 14 days. All animals were sacrificed after 14 days.

### 4.3. Liver Index 

Body weight and liver weight were recorded at the time of the sacrifice. The liver index was calculated using the formula liver weight/body weight × 100.

### 4.4. Histological and Immunohistological Studies

Liver sections underwent staining with hematoxylin and eosin (H&E), Masson, and Sirius red for histopathological examination. Immunohistochemical staining was carried out with glutaminase (GLS1, GLS2)-specific polyclonal antibody.

### 4.5. Biochemical Analyses

Serum samples frozen at −80 °C were thawed until reaching 4 °C and centrifuged at 3000× *g* for 15 min. Serum alanine aminotransferase (ALT) and aspartate aminotransferase (AST) were measured using the ALT/GPT Assay Kit and the AST/GOT Assay Kit, respectively.

### 4.6. Western Blot 

Protein extracts were separated on 10% SDS-polyacrylamide gels and transferred to a polyvinylidene fluoride membrane. The membrane was sealed with non-fat milk for 2 h, incubated with the primary antibody overnight, and then exposed to the subsequent secondary antibodies (1:10,000) for 1h. An ECL chemiluminescence detection kit was used to detect the protein bands. Normalization was conducted based on the level of GAPDH in samples. 

### 4.7. RT-qPCR 

One mL Trizol was added to 50 mg of mouse liver tissue and left at room temperature (15~30 °C) for 5 min. Total RNA was extracted and reverse transcribed into cDNA using a reverse transcription kit under ribonuclease-free conditions with a genomic DNA scavenger. Primer sequences are shown in Table S1. Strip melting curves were analyzed to verify the specificity of the amplification products and SYBR-BASIC was used to quantify the amplification products for gene amplification. The RT-qPCR conditions were the following: 95 °C for 1 min, 95 °C for 20 s for 40 cycles, 60 °C for 45 s for 40 cycles, and 95 °C for 1 min for 40 cycles. β-actin was used as an endogenous control for standardization. The final data were analyzed using the 2^−ΔΔCT^ method [46]. 

### 4.8. RNA Isolation and RNA-Sequencing (RNA-seq) Analysis

RNA-seq of the changes in mRNA profiles between the model group and the ATL III 50 mg/kg group as well as the RNA samples were submitted to UW Genetics Co., Ltd. (Shenzhen, China) for transcriptome sequencing and analysis [47].

### 4.9. Metabolite Extraction and Gas Chromatography–Mass Spectrometry Analysis

One mL of −80 °C pre-cooled methanol solution was added to 50 mg of liver tissue, which was ground and centrifuged at 12,000 rpm for 10 min at 4 °C. The supernatant was transferred to a new tube and dried with nitrogen at 35 °C. The samples were dissolved in pyridine with 80 µL methoxamine hydrochloride (20 mg/mL), centrifuged for 2 min, and incubated at 37 °C for 2 h. Then, 80 µL of bis-trimethylsilyl-trifluoroacetamide (BSTFA) was added, and the sample was centrifuged for 2 min. The samples were incubated in a water bath at 80 °C for 15 min for complete derivatization; then, they were cooled for 5 min, vortexed for 15 s, and centrifuged at 12,000 r/min for 10 min at 4 °C. Finally, 150 µL of supernatant was collected in an injection vial with a liner. Metabolomic analysis was performed using a TRACE 1300 GC-MS system (Temecula, CA, USA), Computer-Aided Similarity Evaluation System for Fingerprinting of Traditional Chinese Medicine (2012 version). A total of 6 samples were collected from each group. Samples were stored at 4 °C and GC-MS analysis was performed within 24 h after preparation. Quality control (QC) samples were prepared by homogeneously mixing 4 μL of solution from each syringe vial into a new syringe vial [48].

The sample injection volume of 1 μL was placed into a separated inlet port equipped with a DB-5MS capillary column (30.0 μm × 250 μm I.D., 0.25 μm film thickness) under the following conditions: the oven temperature was initially maintained at 70 °C for 2 min and then increased to 300 °C at a rate of 5 °C/min and held for 5 min. Helium was used as the carrier gas, the injector split ratio was 10:1, and the constant flow rate was 1 mL/min. The temperature of the injector and mass spectrometer was 280 °C, and the ion source temperature was 200 °C. The energy in the electron ionization mode was set to −70 eV. MS data were acquired in full scan mode in the range of 50–650 mass-to-charge ratio (*m/z*). Metabolomic analysis was performed using the TRACE 1300 GC-MS system (Temecula, CA, USA) along with the computer-aided Similarity Evaluation System for Chromatographic Fingerprint of TCM (Version 2012), Origin software (Version 2018), and SIMCA software (Malmo, Sweden). A total of six samples were collected from each group [49].

### 4.10. Principal Component Analysis (PCA)

PCA was used to compare the differences between the two groups. PCA was implemented using the fast. Prcomp function of the R language and then visualized with the ggplot2 package. 

### 4.11. Partial Least Squares Discriminant Analysis (OPLS-DA)

OPLS-DA was applied to exclude potential confounding variables unrelated to within-group differences and to assess the statistical significance of these signals.

### 4.12. Kyoto Encyclopedia of Genes and Genomes (KEGG) Pathway Enrichment Analysis

The differentially expressed genes from the model group and ATL III 50 mg/kg group were introduced into Metascape (https://metascape.org/gp/index.html (accessed on 6 January 2022)) for the KEGG pathway annotation and enrichment analysis.

### 4.13. Molecular Docking

The binding mode between ATL III and potential proteins was assessed using Auto Dock 4.6.2 to evaluate the binding energy and interaction mode between ATL III and the target protein. The molecular structure of ATL III (CAS: 73030-71-4) was retrieved from the PubChem Compound (https://pubchem.ncbi.nlm.nih.gov/ (accessed on 6 January 2022)). The crystal structure data of PI3K (PDB ID: 4f1s), AKT (PDB ID: 1unp), and GLS1 (PDB ID: 6Iox) were first obtained from the Protein Data Bank (https://pubchem.ncbi.nlm.nih.gov/ (accessed on 6 January 2022)). For docking analysis, all protein and molecular files were converted into PDBQT format with all water molecules excluded and polar hydrogen atoms were added. In the final result, the binding mode with the lowest free energy was selected for the analysis. The 3D model was constructed using pYMOL 1.0.0.0.

### 4.14. Protein–Ligand Interaction Profiler (PLIP)

PLIP (https://plip-tool.biotec.tu-dresden.de/plip-web/plip/index (accessed on 6 January 2022.)) was used to fully automate detection and visualization of relevant non-covalent protein–ligand contacts in 3D structures [50]. The procedure is as follows: enter the ATL III -PI3K, ATL III -AKT, and ATL III -GLS1 complexes from the PDB format after docking.

### 4.15. Cell Culture

LX-2 cells were cultured in DMEM medium containing 10% fetal bovine serum and 1% penicillin–streptomycin at 37 °C with 5% CO2. LX-2 cells were inoculated in 96-well plates at 8 × 10^3^ cells per well and the effect of ATL III on cell viability was assayed using CCK-8 reagent mixed with cells. The LX-2 cells were inoculated in culture dishes and the mod group was activated by adding 5 ng/mL of TGF-β. We determined the dosing concentrations for in vitro cellular assays via CCK8 assay. We found no significant toxicity of ATL III to cells up to 40 μM via CCK8 assay, so we selected 10, 20, and 40 μM as the dosing concentrations for in vitro cellular assays. The drug administration group was additionally treated with 10, 20, and 40 μM ATL III for 24 h. In the con group, only the fluid was changed.

### 4.16. Statistical Analysis

Statistical analysis was performed using GraphPad v8.0. The Shapiro–Wilk test was used to evaluate the normal distribution and the chi-square test was also used. One-way analysis of variance (ANOVA) was performed on normally distributed data, and the results are shown as mean ± standard deviation (SD). The analysis of samples with non-normal distribution or uneven variance was performed using Kruskal–Wallis, and the results are shown as median (quartiles). All data were analyzed using the above method except for RNA sequencing. RNA sequencing results were further analyzed using Dr. Tom Online software from UW Genetics Ltd. (version 2.0). A value of *p* < 0.05 was considered statistically significant.

## 5. Conclusions

**Figure 9:**
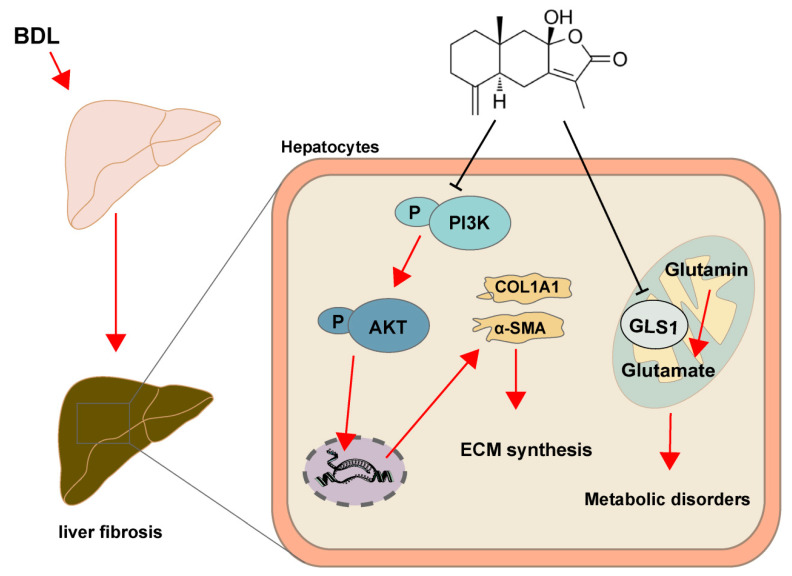
ATL III affects ECM synthesis through the PI3K/AKT signaling pathway, regulates glutamine metabolism to inhibit GLS1, and improves metabolic disorders, thereby ameliorating liver fibrosis.

In conclusion, our results suggest that ATL III ameliorated BDL-induced liver fibrosis by inhibiting the PI3K/AKT signaling pathway, as well as regulating the glutamine metabolic pathway in the liver, providing a reference for the clinical treatment of liver fibrosis (Figure 9).

## Data Availability

The data presented in this study are available in the article.

## Supplementary Materials

The following supporting information can be downloaded at: https://www.mdpi.com/article/10.3390/molecules28145504/s1, Table S1: The sequences of primers; Table S2: Interactions between ALT III with PI3K; Table S3: Interactions between ALT III with AKT; Table S4: Interactions between ALT III with GLS1.
