# Supplementary material for: Atractylenolide III Ameliorates Bile Duct Ligation-Induced Liver Fibrosis by Inhibiting the PI3K/AKT Pathway and Regulating Glutamine Metabolism
Source: Molecules. 2023 Jul 19;28(14):5504. doi: 10.3390/molecules28145504 (PMC10383814; doi:10.3390/molecules28145504)
Supplement: Supplementary file 1 [file molecules-28-05504-s001.zip › molecules-2477594-supplementary.pdf]

## Supplementary Table S1. The sequences of primers.

| Gene name | Forward sequence | Reverse sequence |
|---|---|---|
| Mus-α-SMA | GTACCCAGGCATTGCTGACA | GCTGGAAGGTAGACAGCGAA |
| Mus-COL1A1 | TTCTCCTGGCAAAGACGGAC | TTCTCCTGGCAAAGACGGAC |
| Mus-COL4A2 | CTGGTGAAGCACAGCCAAAC | GTCACCCGGATTGCAGTACA |
| Mus-β-actin | GCAGGAGTACGATGAGTCCG | ACGCAGCTCAGTAACAGTCC |
| Human-β-actin | GACAGTCAGCCGCATCTTCT | GCGCCCAATACGACCAAATC |
| Human-α-SMA | CCTATCCCCGGGACTAAGAC | CCATCACCCCCTGATGTCTG |
| Human-COL1A1 | AAGCTGGAAAACCTGGTCGT | AGCACCATCATTTCCACGAG |
| Human-COL4A2 | GGACAGACGAGACAACAGCA | GAGCTGGCATAACATTGGCG |
| Human-TIMP1 | TCTTGGTTCCCTGGCGTACTCT | GTGAGTGTCACTCTCCAGTTTGC |
| Human-TIMP2 | AGCCAAAGCAGTGAGCGAGAAG | GCCGTGTAGATAAACTCGATGTC |

## Supplementary Table S2: Interactions between ALT Ⅲ with PI3K.

| *Hydrogen Bonds* | | | *Hydrophobic Interactions* | | | *Salt Bridges* | | |
|---|---|---|---|---|---|---|---|---|
| **Residue** | **AA** | **Dist(H-A/D-A)** | **Residue** | **AA** | **Dist** | **Residue** | **AA** | **Dist** |
| 846A | GLN | 2.90/3.29 | 201A | TRP | 3.46 | 849A | ARG | 4.90 |
| | | | 201A | TRP | 3.77 | | | |
| | | | 657A | LEU | 3.74 | | | |
| | | | 694A | PHE | 3.84 | | | |
| | | | 694A | PHE | 3.81 | | | |

## Supplementary Table S3: Interactions between ALT Ⅲ with AKT.

| *Hydrogen Bonds* | | | *Hydrophobic Interactions* | | | *Salt Bridges* | | |
|---|---|---|---|---|---|---|---|---|
| **Residue** | **AA** | **Dist(H-A/D-A)** | **Residue** | **AA** | **Dist** | **Residue** | **AA** | **Dist** |
| 14A | LYS | 3.04 | 17A | GLU | 3.76 | 14A | LYS | 3.75 |
| 17A | GLU | 2.99 | 18A | TYR | 3.79 | 25A | ARG | 3.96 |
| 17A | GLU | 3.85 | 18A | TYR | 3.64 | | | |
| | | | 23A | ARG | 3.34 | | | |

## Supplementary Table S4: Interactions between ALT Ⅲ with GLS1.

| *Hydrophobic Interactions* | | | *Salt Bridges* | | |
|---|---|---|---|---|---|
| **Residue** | **AA** | **Dist** | **Residue** | **AA** | **Dist** |
| 493D | PRO | 3.74 | 543C | ARG | 5.23 |
| 533C | LEU | 3.79 | | | |
